# The Biographical Dictionary of the Society for the Diffusion of Useful Knowledge

**Published:** 1845-04

**Authors:** 


### Art. XIII.

*The Biographical Dictionary of the Society for the Diffusion of Useful Knowledge.* AA—AZ, Vols. I-IV. Seven Parts.—*London,* 1842-4. 8vo.

We peruse with peculiar interest and avidity the biography of those whom we have known, esteemed, and loved. Every incident in their lives is fraught with interest. Their habits, and peculiarities, their dispositions, and feelings,—their domestic concerns, and changes of fortune,—their joys and sorrows, have all a special and direct claim upon our sympathies, and are hallowed in our minds by the most tender associations. The biography of those who have occupied a prominent place in the attention and adulation of our times, is fraught with an interest of a different kind. We strive to discover in the events of their lives or the peculiarity of their minds, the secret of their fame and fortunes. To those who tread the same path to fortune or renown the biography of its heroes is especially instructive. They read in each history the habits of study or reflection, the modes of action and the personal qualities which distinguished its subject, and measuring their strength with his, they emulate the same qualities by which he was characterized, and pursue the same course towards an honorable fame.

These are the objects to which special biography is subservient, and the design for which it ought and must be constructed. Far otherwise is it with such a work as that which we have under review.—It can neither render itself subservient to the gratification of private and personal feelings, nor to the study of human nature in the delineation of the varieties and workings of individual minds; nor can it impart those great moral lessons of instruction and encouragement under difficulties, or of emulation of the virtues and labours of the great, which it is the grand design of biography to convey.

There is another aspect in which biography is peculiarly interesting and instructive, namely, as presenting a history of the great events, or scientific discoveries with which the individuals whose lives it records were connected. In this manner the history of a great statesman becomes the history of the political events of his times,—that of a series of emperors becomes the history of their empire. So may the history of a series of scientific men, if arranged with a reference to their respective sciences, and the successive ages in which they lived, become a history of chemistry, or of medicine, or of any other art or science. This end of biography can be but imperfectly, if at all, attained in a biographical dictionary where the lives are arranged without reference either to the time or place at which the individuals flourished, or the qualities or achievements by which they were distinguished. The design of such a work is to put on record the names of all persons whose names deserve to be recorded, whatever the amount of their desert or the nature of the acts or excellencies for which they were celebrated. Their names must be arranged in the most convenient form for reference, without regard either to their comparative merits, the age or country in which they lived; the arts or sciences which they cultivated; or, in fine, the reasons for which their names have been enrolled in the book of fame. The aim of a general biographical dictionary must be to comprise as many names as are worth knowing,—to give a few particulars regarding the period in which the persons lived, their country, the titles of their works, and such references as may enable the individual who consults it to obtain more complete information regarding the persons and their works. It is in short a book of reference, the design of which is to enumerate the leading facts regarding the persons whose names it comprises, and to indicate the sources of further information regarding them.

For convenience, in consulting such a work, the names of the persons included in it are arranged in alphabetical order. This mode of arrangement, while it possesses many obvious advantages, is not without many objections which might be urged against a work the principal or sole design of which must be that of reference. It presupposes that, before the work can be consulted, the name of the individual we wish information concerning, must be known. Now, in nine cases out of ten in which we wish the assistance of such a book of reference, to know the *name* of the individual is the very thing we want; if we know the name, we already possess the means of obtaining such further sources of information regarding him, as such a work as the present can offer. A biographical dictionary may suffice to afford us the means of determining readily the date of an emperor's birth or death, the place where a poet was born, or the titles of all the works of a distinguished author. But the sort of information, most of all required by the student, is of an opposite kind,—it begins with the events, or subjects of inquiry, and ends with the actor or author of them. The questions regarding which he requires the aid of a book of reference are such as these; *who* has written on such a *subject*,—*who* has written the history of such a period, or country,—*who* has prosecuted investigations in such a branch of science? When he knows the *names*, any library catalogue will in most instances furnish him with the works required, and indicate all the sources of information he stands in need of.

The relation of biography to one of the objects referred to in the preceding remarks is adverted to in the preface of the work before us, and the means of obviating the defect incidental to an alphabetical arrangement of such materials, as far as regards that object, is promised to be provided for.

"A biographical dictionary," says the accomplished editor, "may be used for other purposes than that of merely referring to it for individual lives. The lives of men who were contemporary and in certain relations to one another, as political personages, teachers of philosophy, and writers generally, or the lives of personages who are in a certain relation of succession to one another, as kings of the same dynasty, may be selected out of the alphabetical order, and so read for the purpose of comparison, or for the purpose of combining the information contained in several lives, that is, for the purpose of historical study. In order to facilitate this use of the dictionary, the last volume will contain tables of kings and other public personages, who are related to one another in the order of succession; and it will also contain certain synchronitic tables, which will exhibit in their relations of time those personages who have had the chief influence on the course of human affairs, and on the progress of knowledge." (Preface, p. vii.)

Such tables will enable the readers to peruse a series of lives in their chronological order, and thus to make the work a means of historical study either in reference to countries or great discoveries. So far it will greatly enhance the value of the work, and will make it in other respects more available as a book of reference. The value of the work would be still more increased if such tables were extended to all the persons whose names are recorded in the Dictionary. Dividing them into different classes according to their rank or profession, or other distinctive characters, and arranging them in chronological order.

If, in addition to such tables, the Society were to publish as a supplement, a *catalogue raisonné* of all the works mentioned in the biographies, they would render a most notable service to literature and science. A few such catalogues we possess, but they are mostly of theological works, or limited to other departments of study, and the best of them are far from being so complete as they might be, besides being so rare or expensive as to place them, in a great measure, beyond the reach of the student. The Dictionary before us promises to be the most elaborate and complete work of the kind ever published. Attached to each biography is a list of the titles of all the works published by the individual, if an author. Such a catalogue therefore might be readily compiled out of the Dictionary itself, after its completion. The catalogue would probably extend to several volumes, and would be divided into different parts according as the works related to theology, law, history, travels, the arts or sciences, poetry or general literature; and these again would necessarily require to be subdivided according to the different departments of each subject to which the works severally referred. Such a catalogue would render the Dictionary in every respect complete as a book of reference, and would, in itself, be a work of unspeakable advantage to every student.

The seven parts of the Dictionary now published comprise all the names under the letter A. The work appears to have been executed with great labour and accuracy. It is chiefly valuable for its completeness,—one of the principal objects undoubtedly in such a work; and certainly the one before us, leaves nothing apparently to be wished for in this respect. The number of names must indeed appear astonishing to most readers. No labour or research seems to have been spared in making the notices of every individual as accurate as possible; and especially, what must render the work peculiarly valuable, in giving such references to the writings of the individual and all the sources of information regarding him as will satisfy all those who consult it for ulterior purposes.

It would be beyond our province, if we were qualified to pass a judgment on the mode in which all the biographies have been written. It appears to us to be peculiarly rich in the historical and theological departments. This may arise from our ignorance of those departments, more particularly the latter; but certainly the number of Aarons and Abrahams is, to our medical eyes, something more than astonishing. Every department, however, seems to be justly and equally dealt with, and the extent of each biography to bear a fair relation to the importance of the individual.

We are certainly entitled to pass our opinion upon the medical biographies, of which a brief survey of a portion may interest our readers, and afford a good sample of the general merits of the work.

There are not less than 250 lives of medical men in the volumes before us; only a few of them, however, have any particular claim upon our attention, or that of the general reader; although, doubtless, they have each their special interest in relation to their respective epochs, or to particular subjects of inquiry.

The first physician occurring in the alphabetical arrangement of the Dictionary is Pietro di Abano, or Petrus Apono, a professor of medicine, and physician in Padua, in the beginning of the fourteenth century. Like most of the medical writers of that period, he is known to us only as a commentator upon the fathers of medicine, and the physicians of the Arabian schools. His principal work is entitled 'Conciliator Differentiarum Philosophorum et precipue Medicorum;' from which he received the name of *Conciliator*. With many of his contemporaries he partook in a large amount of credulity in relation to astrology and magic, and incredulity in regard to points of religious faith. For these offences he was tried before the Inquisition once, and again, and ultimately died in 1315, while his second trial was going on. Abano was distinguished by a work on Poisons and their Remedies.

Of Abascantius, the next physician in the Dictionary, all that we know with certainty is, that he invented an antidote against the bite of serpents, referred to several times by Galen.

The next physician we come to, Baldi Angelo Abbatio, physician to Francesco Maria II, Duke of Urbino, in the early part of the sixteenth century, is also known to ns in connexion with the same subject, by his work 'De Admirandâ Viperæ Naturâ.'

The two next in order are ABDU-L-LATTIF and ABDU-L-MALEK IBN ABHAR ALKENANI. The latter was a Christian physician and professor of medicine at Alexandria, who is said to have embraced Islám about the year 689. The former was a celebrated Arabian physician, historian, and traveller of the twelfth and beginning of the thirteeeth centuries, who rejoiced in the names, as given at length, by Wüstenfeld, of *Abû Mohammed Abdu-l-lattif Ben Yusef Ben Mohammed Ben Alí Ben Abí La'd Ibnul-lebad Muwafiku-d-din al-Baghdâdi!* He studied at Bagdad, and held professorships at Mosul, Damascus, and Cairo successively. He also obtained much reputation as a practical physician, as well as lecturer, both at those places and at Jerusalem, Aleppo, and in Asia Minor, where he travelled. "Like most of the great Arabic writers, Abdu-l-lattíf appears to have been a very voluminous anthor. Ibn Abí Ossaybíah has preserved the titles of 166 works written by him, of which nearly one fourth related to medical subjects; but of these only one has been published or translated, the 'Compendium of the History of Egypt.' This is an abridgment, by himself, of a larger work on the same subject, and consists of two parts. The former contains six chapters, treating, 1, of the general character of Egypt; 2, of plants; 3, of animals; 4, of the ancient monuments; 5, of buildings and ships; 6, of the food of the Egyptians. The second part contains three chapters, treating, 1, of the overflowing of the Nile; 2, 3, of the years (A.H. 597, 598), (A.D. 1200, 1201); i. e., the years of the famine." The work is chiefly interesting for the account of this famine, which occurred while he himself was in Egypt. The horrors of it appear to have equalled or exceeded "anything of the kind in ancient or modern times."

"The famine was occasioned by the Nile not having risen to its usual height the year before, and it extended over the whole of Egypt. Besides the vices and crimes that usually prevail during such calamities, the extent to which human flesh was eaten was almost incredible. Children and grown-up persons were seized and entrapped in all sorts of ways, and the food which was at first eaten from necessity grew at last so palatable, that it was cooked with spices and sauces of various kinds, and considered as a delicacy. The instances of children being murdered and eaten by their own parents were so frequent, that though the crime was punished by burning the offenders alive, it could not be prevented; and the nearest relatives claimed as a right the bodies of their friends, saying that it was fitter they should be eaten by them than by strangers." (Vol. i, part i, p. 77.)

The biography of Abdu-l-lattif contains some further particulars regarding the life and writings of that physician, and ample information concerning the different editions, Arabic, Latin, German, and French, of his great work. Of these the most valuable is a French translation, published in 1810, by De Sacy (Paris, 4to), enriched by critical and explanatory notes, in which he was assisted by the most eminent zoologists and botanists of Paris.

The above sketch of the biography of the first physician occurring in the Dictionary of any celebrity will afford a fair specimen of the mode in which the biographies are generally executed throughout the work. The kind of information communicated is most valuable, and exactly that for which such a work is likely to be consulted, embracing the dates of the individual's life and death, the principal incidents in his history, a notice of the works by which he was known, with a brief summary of their contents and character, and such references as will enable any one to obtain full information regarding the individual, or to consult or furnish himself with his writings.

A very brief notice of SCIPION ABEILLE, a French military surgeon, distinguished by the eccentricity of his works, in which he endeavoured to render anatomy interesting, by combining it with poetry; and of Dr. CLARKE ABEL, the physician, naturalist, and historian of Lord Amherst's embassy to China; is followed by short biographies of DAVID and PATRICK ABERCROMBY, Scottish physicians of the seventeenth and early part of the eighteenth centuries. These contain also ample references to the works of the authors respectively, and to the sources of further information regarding them. The writings of David Abercromby have not yet altogether lost their interest, particularly his treatise 'De Variatione ac Varietate Pulsus,' which is characterized by great acuteness of observation, ingenuity, and that sort of originality which prevents a book from ever becoming too old to be read.

These biographies bring us to our own times in the next physician in the alphabet, JOHN ABERNETHY, the second "glorious John" of our literary history. The sketch of his history, opinions, and talents, and the influence which he exercised on the surgical profession in this country—the critique on his writings, and the estimate of his abilities as a lecturer, are all written with much ability, and, in our opinion, sound judgment. It is followed by a complete list of Mr. Abernethy's writings, extending through an entire column, and comprising also a brief notice of their contents and merits.

Passing over a number of learned physicians of less note, Danish, Jewish, Portuguese, Arabian, Italian, German, and Swedish, we come, at p. 242 of the same volume, to ACHILLINI, professor of medicine at Padua and Bologna in the early part of the sixteenth century, a name which deserves to be ever remembered as that of the earliest to revive the study of anatomy by actual observation and dissection of the human body.

"His anatomical descriptions, though chiefly collected from the writings of Mundinus and the Arabian physicians, yet contain accounts of several things before unknown, as the nerves of the fourth pair, the number of the bones of the tarsus, and the position at which the spinal cord terminates; and exhibits a more accurate knowledge than his predecessors had of many other parts, such as the olfactory nerves, the veins of the arm, &c. He is also commonly said to have discovered the two bones of the ear named malleus and incus; but he does not claim the honour, and there is no evidence to prove more than that they were discovered in his time."

The titles of the philosophical and medical works of Achillini are very fully given in the biography before us.

The last biography we shall notice is that which follows next in order, the biography of J. F. ACKERMANN. It is clear, succinct, and comprehensive, and affords an excellent specimen of the merits and utility of the whole work, as far as it relates to the medical profession. After noticing his birth, education, travels; the professorships which he held at Mainz, Jena, and Heidelberg; the improvements which he effected in the system of medical education, and his death, in 1813,—the following abstract is given of his writings and opinions. These are interesting in relation to the direction which similar inquiries and theories have taken of late years.

"J. F. Ackermann's chief works are those in which he attempts to supplant the current physiological theory of a vital principle, by one in which all the processes of the living body were made to depend on the chemical relations between the organized tissues and oxygen. He supposed 'that the vital movements are nothing but the alternate contractions and dilatations of the organic tissue and the cells which compose the mechanical elements of the organs, and that these movements depend on the slow combustion which goes on between oxygen and the materials, as well fluid as solid, of the organic body. (*Kleineren Schriften*, s. 163.) The first stage of this slow combustion was thought to be effected in respiration, in which the oxygen combined with caloric, so as to form a kind of aura, or *semi gas*, and being received into the blood, united with certain principles of it, as gelatine, albumen, &c. Rendering the latter solid, as he imagined, it formed the blood-globules, and as it passed along the vessels that convey red blood, oxidised all the tissues with which it came in contact, making them at the same time contract so as to carry on the circulation of the blood. The essence of nutrition was made to consist in the particles of blood impregnated with oxygen-aura (or, as he sometimes calls it, *vital ether*), permeating the walls of the vessels, and becoming solid; and in the refuse particles of the tissues, in which the combustion was completed, assuming the fluid form, and entering the lymphatics. And this chemical action he regarded as the origin of a mechanical force; for as the oxidised parts, when they are made fluid, separate from the cellular tissue (of which all organized substances are composed), the walls of the cells contract, force on the fluid with which they are charged, and are in their turn again dilated, when the particles to replace those that have separated are inserted into them. This constitutes the *automatic irritability;* and through this, by the constant action of the oxygen-aura in the blood upon the walls of the left heart and arteries, the circulation is maintained. The same aura is separated from the blood-vessels of the brain, and stored up in that organ, and employed through the medium of the nerves to produce a more intense and prolonged slow combustion of the carbon from the cells of the muscles, for the production of their contractions, or, as he supposed, their actual condensation.

"It is unnecessary to enter into the details of this hypothesis; and it would hardly have merited any notice, had it not been among the first steps by which later physiologists were slowly brought to acknowledge that the essence of what is called a vital process does not consist in its being utterly different from those observed in dead bodies; but rather, that many of the processes which go on in the living body are only very complex examples of the influence of the same forces which operate in dead matter. Defective and erroneous as Ackermann's physiological system was, yet since he gave to oxygen both the name and the dignity of the 'principle of life,' he set many on the search after the real influence of that element upon the economy, and what was yet more important, he shook the common faith in the less definite but hardly less erroneous systems of his predecessors.

"The following are the titles of Ackermann's chief works, and they will sufficiently indicate the contents of those which do not relate to his hypothesis:—'Dissertatio de Discrimine Sexuum præter Genitalia.' This is better known in the German translation by Jos. Wenzel, Koblentz, 1788, 12mo.—'De Nervorum Opticorum inter se Nexu,' published in Blumenbach's Bibliotheca Medica, iii, 337.—'Ueber die Kretinen;' Gotha, 8vo, 1790.—'Versuch einer physichen Darstellung der Lebenskrafte organisirter Körper,' Frankfort on the Main, 2 vols. 8vo, 1797-1800.—'Infantis Androgyni Historia et Ichnographia,' folio; Jena, 1805.—'De Humanæ Naturæ Dignitate, accedit de Nervei Systematis Primordiis Prolusio,' 8vo, Heidelberg, 1811. The last is to establish that the heart is the centre of the nervous system, and that the nerves are in the first instance formed from the blood which filters into its walls in the manner already mentioned. Besides these, he wrote several smaller essays, on fever, on typhus, against phrenology, on the thyroid body, &c.; the best of which were translated by C. Hoffmann with the title 'Sammlung der wichtigsten lateinischen Scriften des J. F. Ackermann,' 8vo, Speyer, 1816; and of which an account may be found in the Almanach der Universität Heidelberg auf das Jahr 1813. (The life of Ackermann has been written by F. Molter, in Ersch and Gruber's Allgemeine Encyclopädie.)" (Vol. i, part i, pp. 246-7.)

We might continue this sketch through the whole seven parts of the Dictionary we have now before us,—but it would far exceed the limits of any Review to notice even the names of the medical men comprised in them. A considerable proportion of them are Greek and Arabian physicians. The third volume contains a most complete and elaborate account of the Appolonians.

We might be tempted to dwell on a few at least of the names which are presented to us—Æsculapius, Alcmæon, Albinus, Alberti, Aldrovandus, Avenzoar, Avicenna, the Armstrongs, Adams, Arnaud, Asellius, Autenreith, the Greek and Arabic physicians and others, but *cui bono?* The object of such a work as this is not to present its readers with the results of antiquarian lore or original research regarding the particulars of the lives of those whose history it briefly records, or to present to us new facts regarding them. Each individual biography does not, therefore, present matter for detailed criticism or review. We conceive that we have done justice to our readers and to the work by stating the plan on which it is constructed, by pointing out the objects which it may subserve, by showing how far these have been kept in view in the specimen before us, and by giving such samples as may enable every one to judge of the extent, ability, learning, and general utility of the work. We might have selected the lives out of the several volumes and viewed them in chronological order—in relation to the progress of medicine,—but such a survey would have been necessarily imperfect from its containing the lives only of a limited number coming under the letter A and would in itself have been subverting, at, great trouble to ourselves, the plan and arrangement of the work, for ulterior purposes.

The very learned editor of this great work is Mr. Long; and among his principal contributors in the class of medical lives are Dr. Greenhill and Mr. Paget. We recommend the work to our readers as one altogether unrivalled in the comprehensiveness of its objects, the originality and completeness of its materials, the fidelity and trustworthiness of its statements, and the general excellence of its execution.

---

